# Supplementary material for: Multistage coupling water-enabled electric generator with customizable energy output
Source: Nat Commun. 2023 Sep 14;14:5702. doi: 10.1038/s41467-023-41371-x (PMC10502115; doi:10.1038/s41467-023-41371-x)
Supplement: Supplementary file 1 — Supplementary Information [file 41467_2023_41371_MOESM1_ESM.pdf]

Supplementary Information

Multistage Coupling Water-enabled Electric Generator with Customizable Energy Output

Puying Li[1], Yajie Hu[1], Wenya He[1], Bing Lu[1], Haiyan Wang[1], Huhu Cheng[1]* & Liangti Qu[1]*

[1] Laboratory of Flexible Electronics Technology, Key Laboratory of Organic Optoelectronics & Molecular Engineering, Ministry of Education, Department of Chemistry, State Key Laboratory of Tribology in Advanced Equipment (SKLT), Tsinghua University, Beijing 100084, P. R. China.

Characterization

Scanning electron microscope (SEM) was acquired by using Hitachi cold-field emission scanning electron microscopes. Energy dispersive X-ray spectra was carried out by the use of an INCA Energy EDS System (Oxford Instruments, UK). X-ray photoelectron spectra (XPS) was taken on an ESCALAB Xi^+ photoelectron spectrometer (Thermo Fisher) with an Al Kα source. Fourier transform infrared spectra (FTIR) was conducted on an UATR Two FTIR spectrometer (Pekin Elmer, USA). Zeta potential was measured by using Zetasizer Nano ZS90 (Malvern Panalytical, UK). Moisture absorption and desorption kinetic was analysized by Dynamic Vapor Sorption (Intrinsic, UK).

Structure and internal water transmission path of mc-WEG

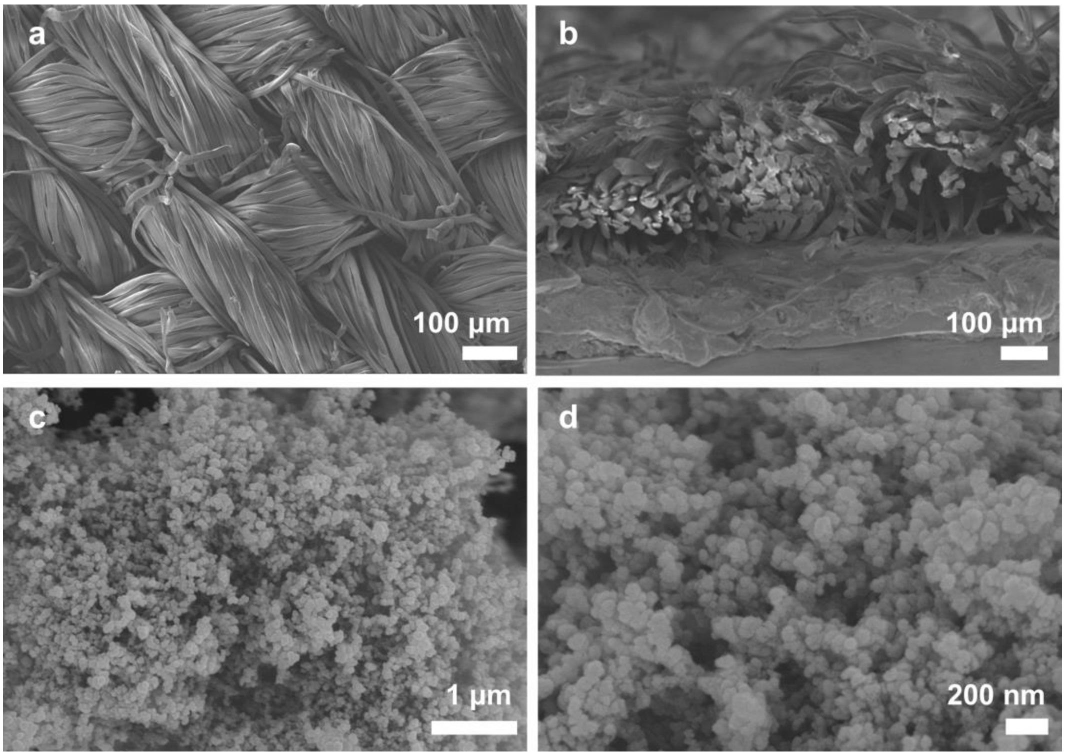

Supplementary Fig. 1 Top (**a**) and cross-sectional (**b**) SEM images of cotton fabric. **c, d** SEM images of carbon black nanoparticles (Ketjen black).

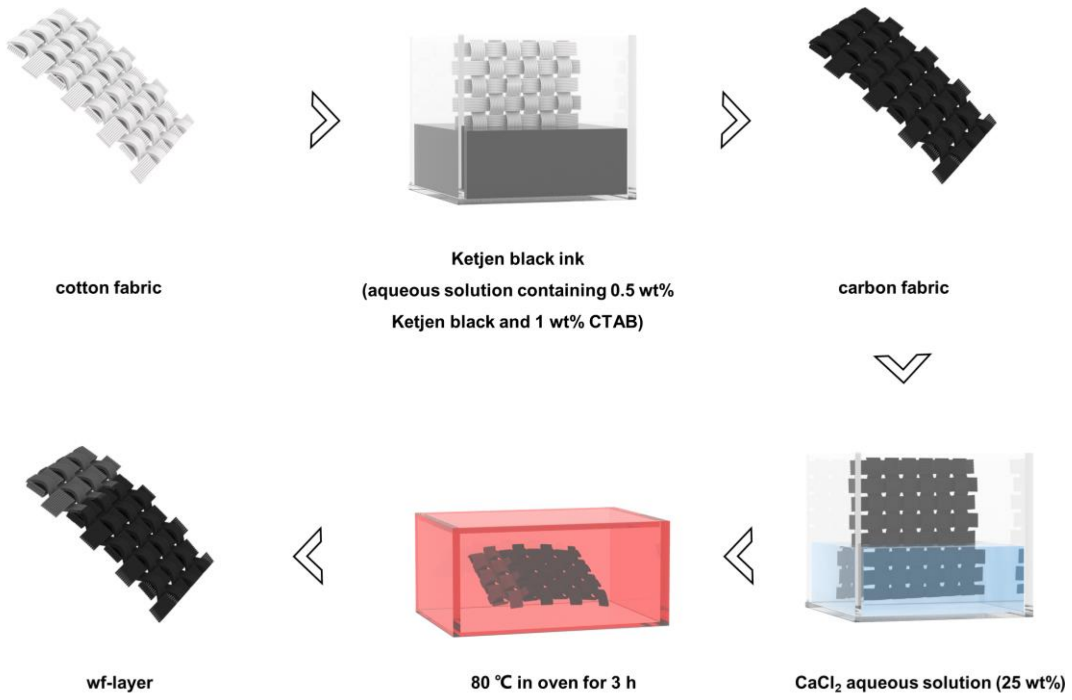

Supplementary Fig. 2 Schematic illustration of the preparation process of wf-layer.

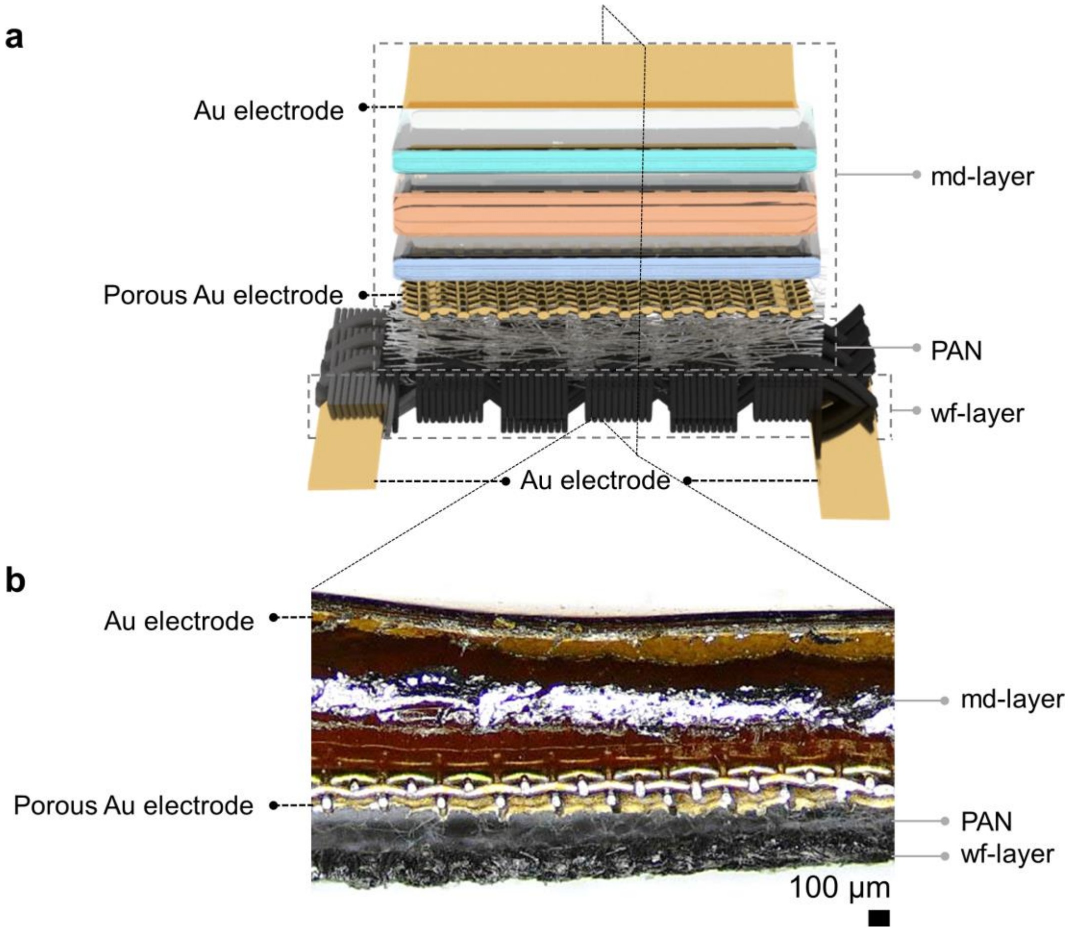

Supplementary Fig. 3 Schematic diagram of the structure of mc-WEG (**a**) and the cross-section digital image of mc-WEG (**b**).

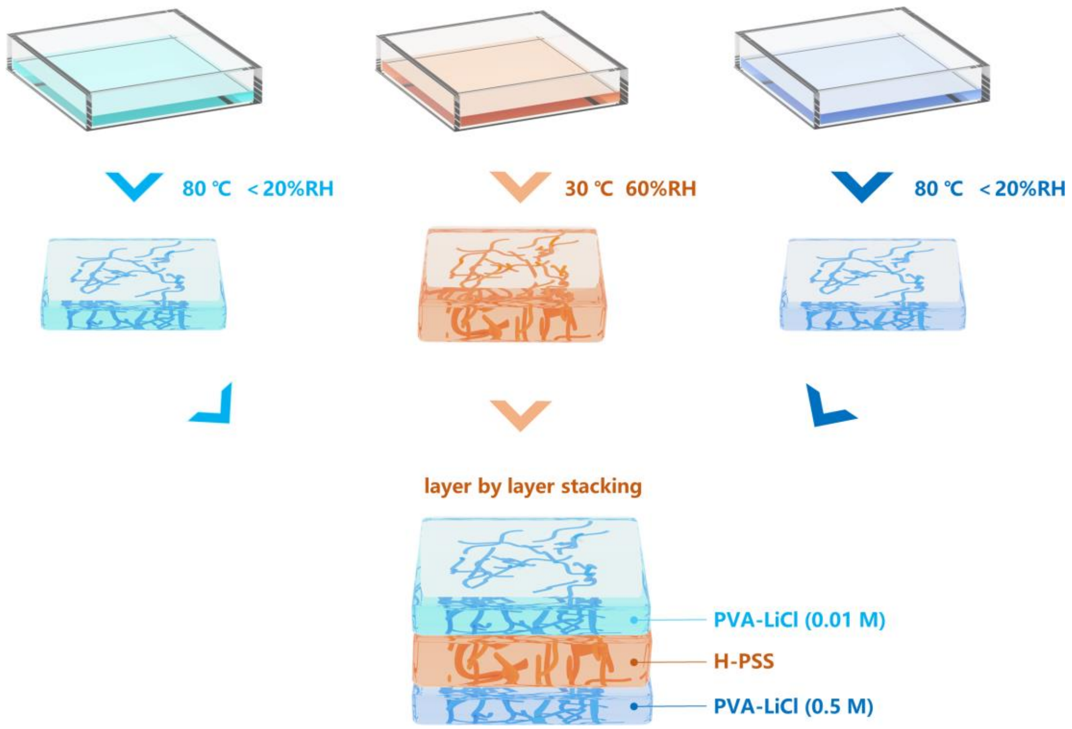

Supplementary Fig. 4 Schematic illustration of the preparation process of md-layer.

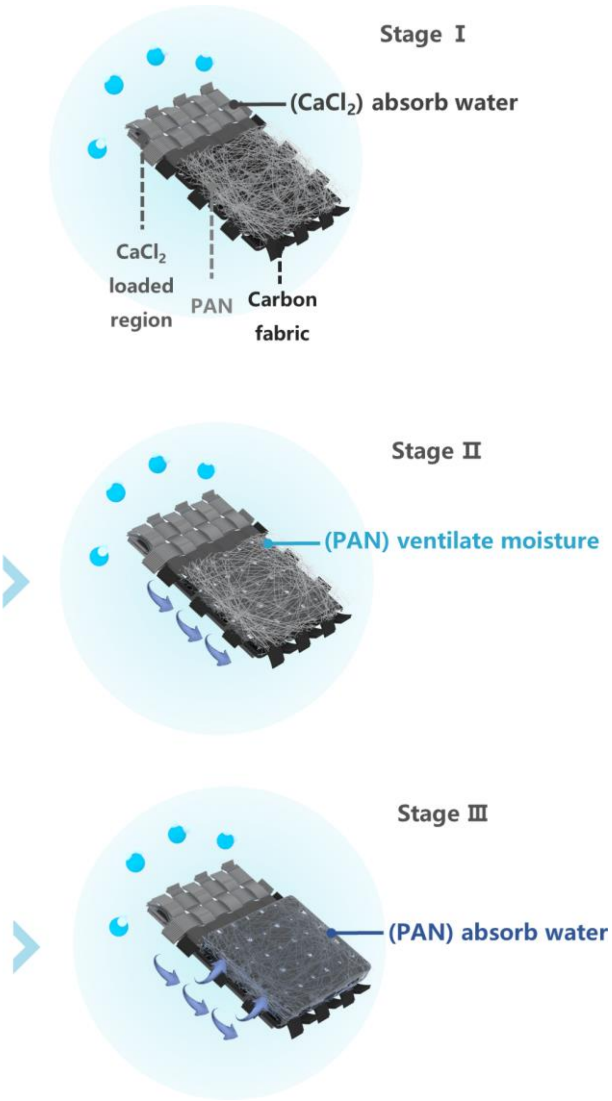

Supplementary Fig. 5 Three stages of water transport in mc-WEG. The navy blue arrows represent the flow direction of liquid.

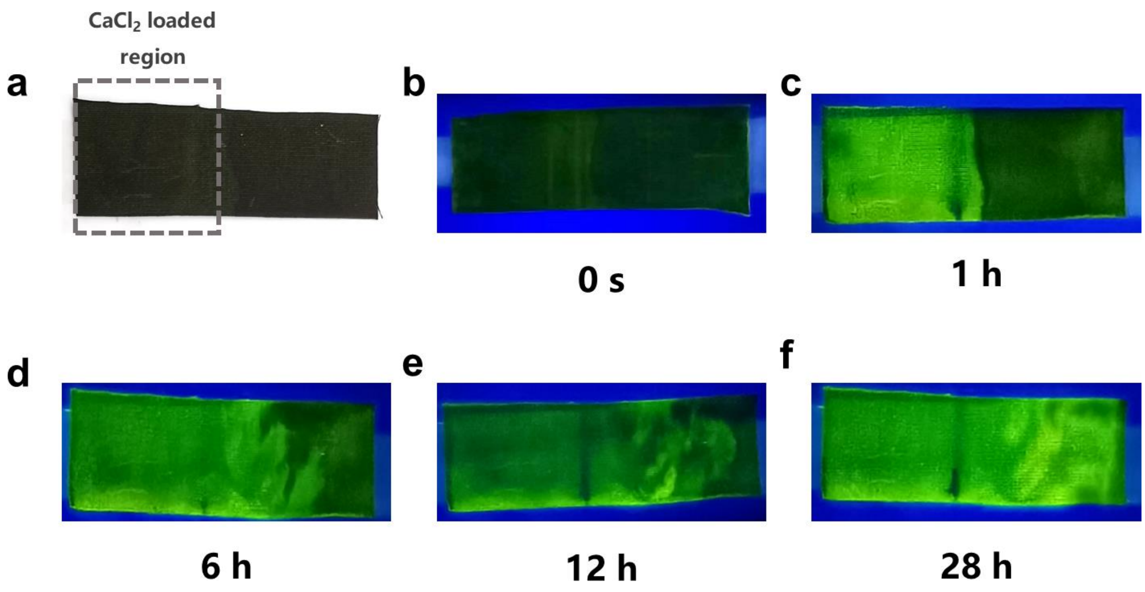

Supplementary Fig. 6 Water transport processes of wf-layer ($CaCl_2$ loaded region was 2 cm × 2.5 cm from left to right). wf-layer was pre-impregnated with sodium fluorescein. Digital photographs under natural light (**a**) and ultraviolet light (**b**) of wf-layer after pre-impregnating with sodium fluorescein. wf-layer was then exposed in moisture (90 %RH) and observed under ultraviolet light at 1 h (**c**), 6 h (**d**), 12 h (**e**) and 28 h (**f**). Green regions are the water infiltrated part.

Electricity generation performance of wf-layer

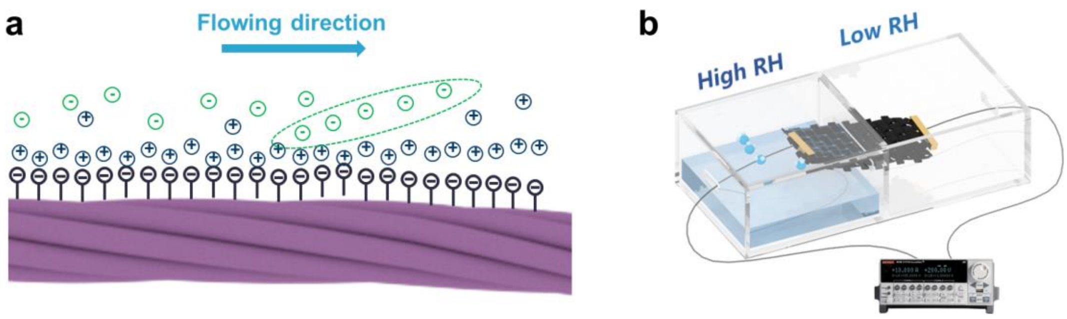

Supplementary Fig. 7 a Schematic diagram of electricity-generating principle of wf-layer. **b** Test diagram (asymmetric humidity environment) of wf-layer.

Supplementary Note 1: Due to the functional groups (carboxyl group, hydroxyl group) and $CaCl_2$ on the carbon fabric, cations will be formed and absorbed on the surface when wf-layer absorbs water from the environment, which will flow along the wf-layer with the flow of liquid. The flowing process caused the unbalanced charge between the anions and cations at the front end of the evaporation side, as the anions are more retarded in migration to form the diffusion layer and counteract the net charge of the stern layer[1,2].

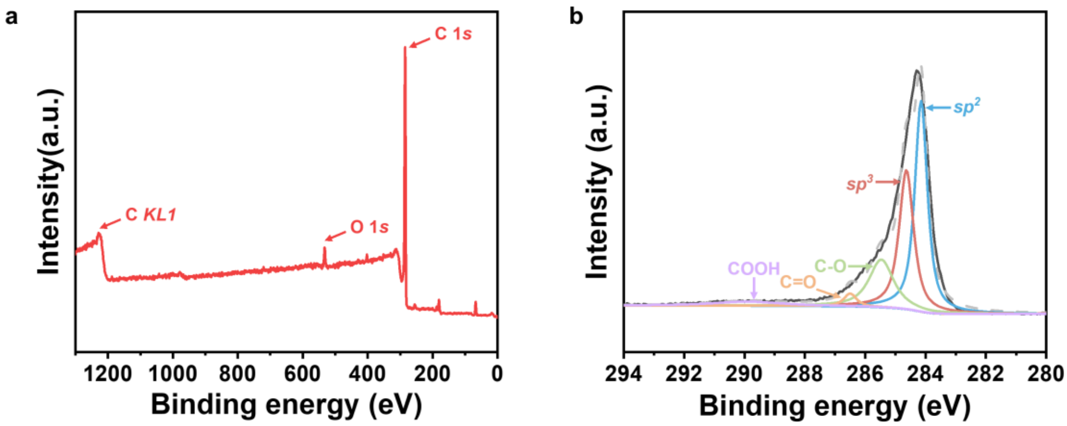

Supplementary Fig. 8 The X-ray photoelectron spectroscopy (XPS) (**a**) and fine spectroscopy for C 1*s* (**b**) of carbon fabric. Source data are provided as a Source Data file.

Supplementary Note 2: XPS analysis was used to analyze the functional groups formed on the surface of carbon fabric. The peaks of hybridized carbon atoms were found at 284.15 (sp^3) and 284.64 eV (sp^2). The peaks of oxygen functional group were found at 285.47 (C-O), 286.50 (C=O), and 289.90 eV (COOH)[3,4].

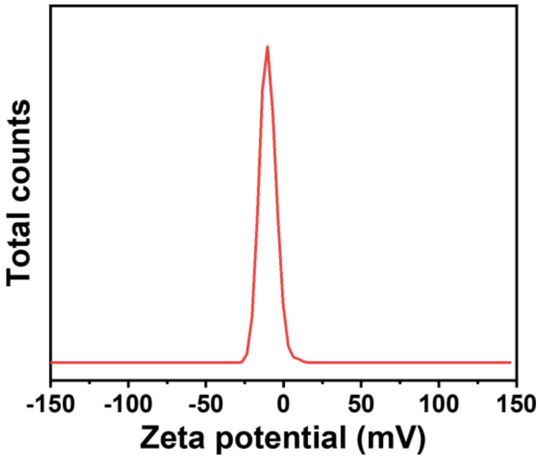

Supplementary Fig. 9 Zeta potential (-10.1 mV) of Ketjen black particles in distilled water. Source data are provided as a Source Data file.

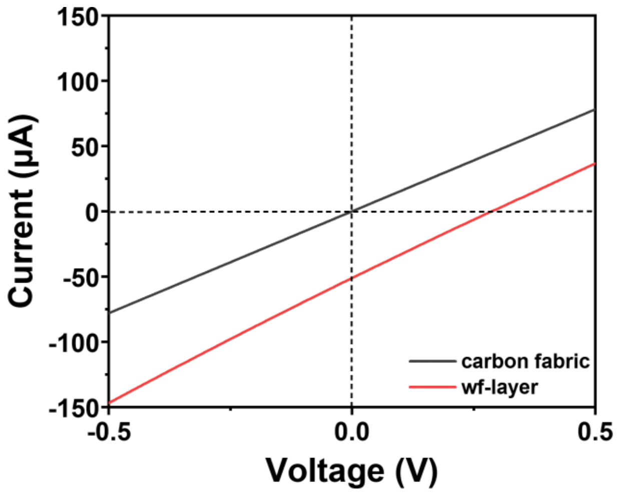

Supplementary Fig. 10 Current-voltage curves of carbon fabric (black line) and the wf-layer (3 cm× 6 cm, red line). Source data are provided as a Source Data file.

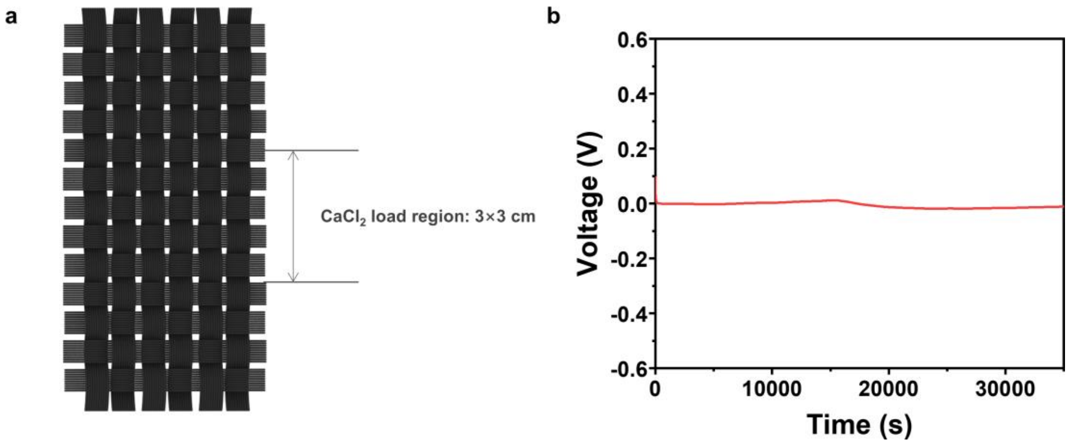

Supplementary Fig. 11 a Schematic diagram of the $CaCl_2$ loaded area of the carbon fabric. **b** Voltage-time curve of carbon fabric (3 cm × 9 cm) loaded with $CaCl_2$ in the middle (~90% RH). Source data are provided as a Source Data file.

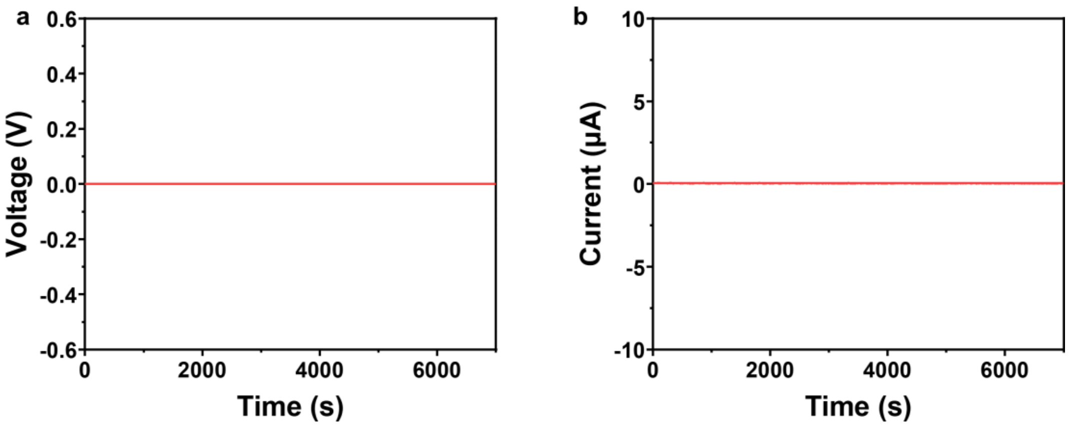

Supplementary Fig. 12 The voltage-time curve (**a**) and current-time curve (**b**) of wf-layers under dry state. Source data are provided as a Source Data file.

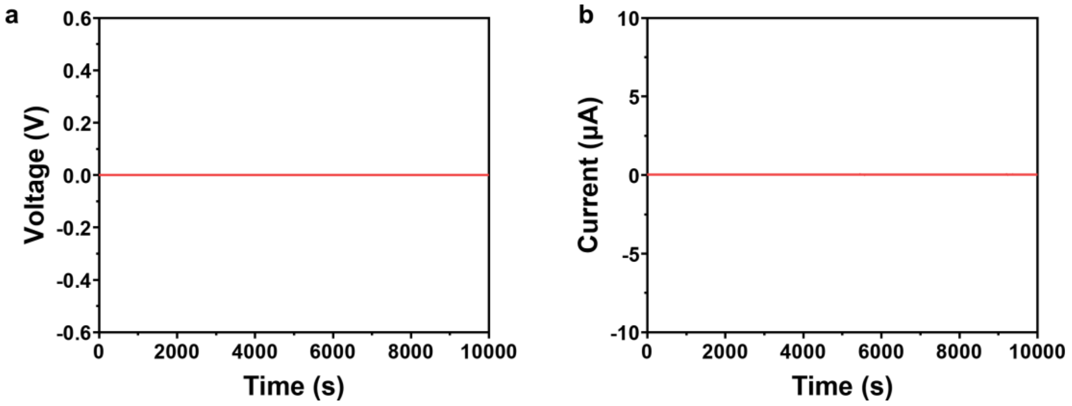

Supplementary Fig. 13 The voltage-time curve (**a**) and current-time curve (**b**) of pure carbon fabric under the test condition of wf-layer. Source data are provided as a Source Data file.

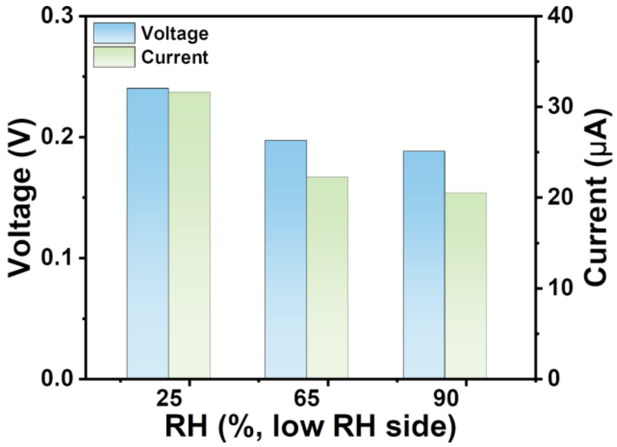

Supplementary Fig. 14 The electric generation performance of wf-layers at different RH on the low RH side. Source data are provided as a Source Data file.

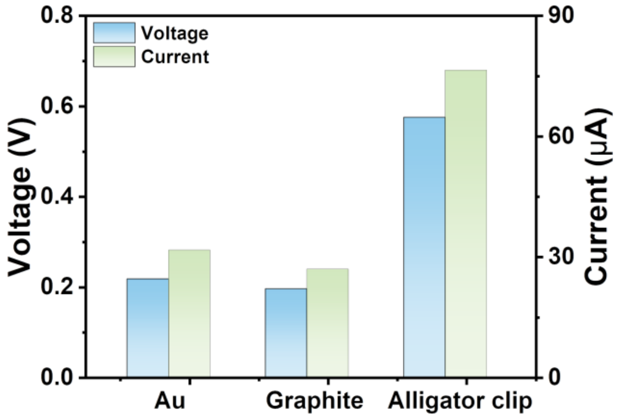

Supplementary Fig. 15 The electric generation performance of the wf-layers (3 cm × 6 cm) with different electrodes (Au, graphite, alligator clip) under asymmetric humidity environment (60% RH for low RH side, 90% RH for high RH side)**.** Source data are provided as a Source Data file.

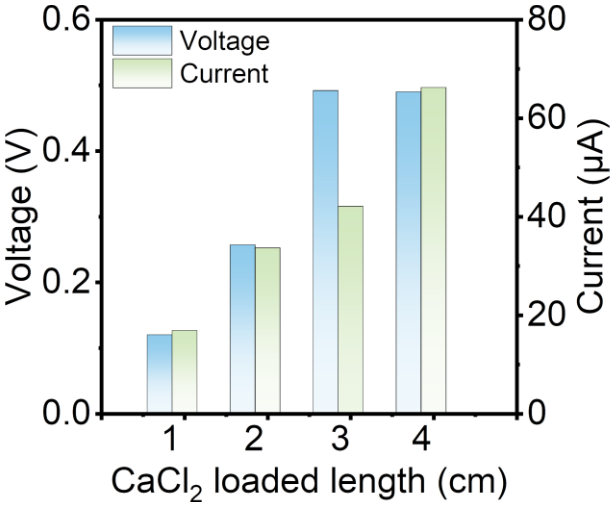

Supplementary Fig. 16 The electric generation performance of wf-layers (3 cm×6 cm) with different $CaCl_2$ loaded length. Source data are provided as a Source Data file.

Supplementary Note 3: As can be seen from Supplementary Fig. 16, the voltage and current increased from 0.12 V to 0.49 V and 16.89 μA to 66.22 μA as the $CaCl_2$ loaded length increased from 1 cm to 4 cm in the wf-layer unit (3 cm×6 cm), because more water could be absorbed by wf-layer with longer $CaCl_2$ loaded length.

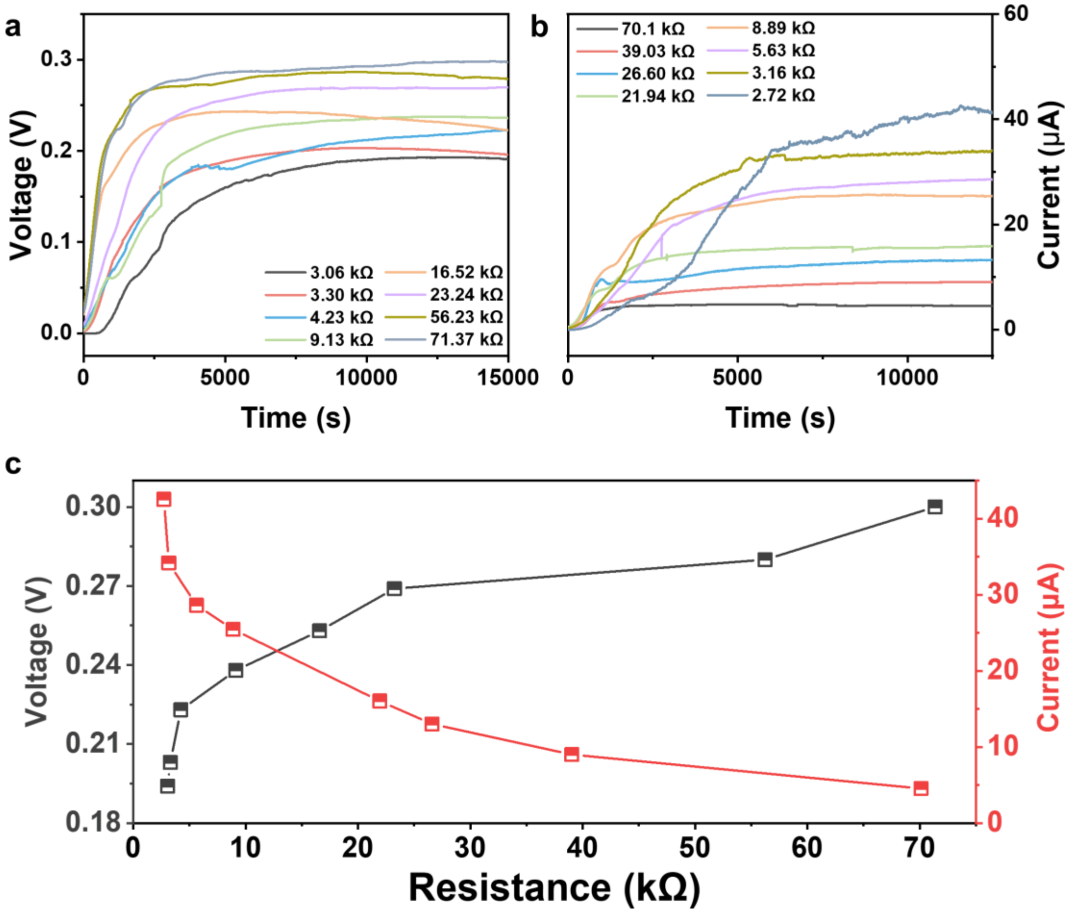

Supplementary Fig. 17 Voltage-time curves (**a**), current-time curves (**b**) and scatter graph (**c**) of wf-layers (3 cm × 6 cm) with various bulk resistance under asymmetric humidity environment (60% RH for low RH side, 90% RH for high RH side). Source data are provided as a Source Data file.

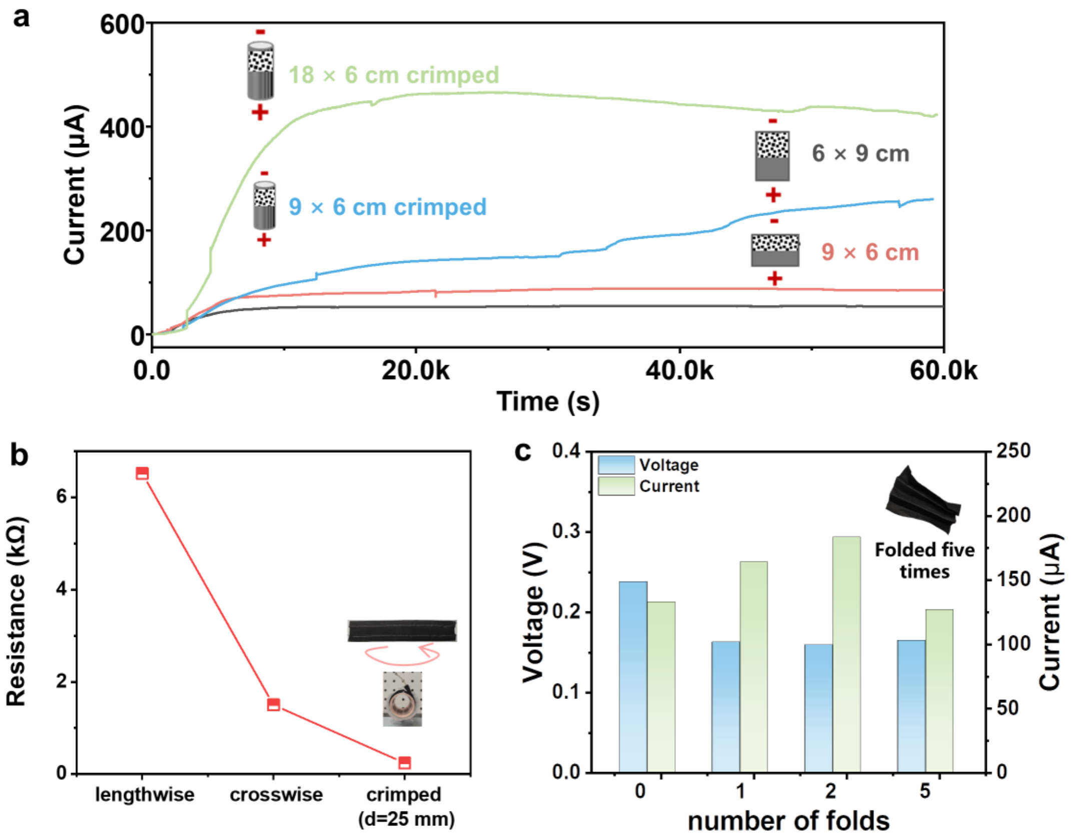

Supplementary Fig. 18 a Current-time curves of wf-layers with different test ends and spatial form. Inset shows the sketch map of spatial form. **b** Resistance of wf-layers (18 cm × 6 cm) under different test ends and spatial structure. Inset shows the digital photo of crimped form. **c** Voltage and current output of wf-layer (6 cm × 6 cm) with different number of folds. Inset shows the digital photo of wf-layer folded five times. Source data are provided as a Source Data file.

Supplementary Note 4: For wf-layers with the same size, the output current is larger when the long end is taken as the output end, and crimp design can further increase the current (Supplementary Fig. 18a) due to the reduced resistance (Supplementary Fig. 18b).

Meanwhile, the generated current of wf-layer (6×6 cm) first enhances and then reduces with the increase of number of folds (Supplementary Fig. 18c). The enhancement can be attributed to the decrease of the resistance, and the subsequent decline is caused by the

reduced water absorption due to close packing. No significant attenuation in generated voltage was observed after the first fold. Meanwhile, for wf-layer with the same size, as the number of folds ascends, it can be considered that the width of wf-layer gradually descends and the number of parallel connections gradually increases:

$$\text{number of parallel connections}=\text{number of folds}+1$$

$$\text{unit's width}=\frac{\text{wf-layer's width}}{\text{number of parallel connections}}$$

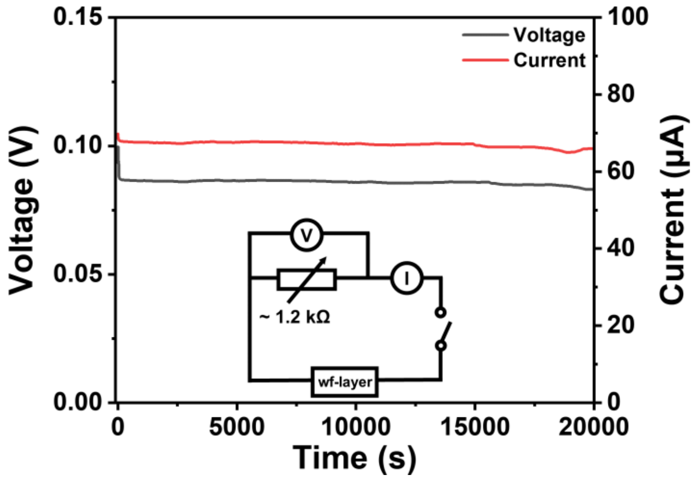

Supplementary Fig. 19 The voltage and current output supplied by wf-layer (18 cm × 6 cm, crimped state) connected with resistance of 1.2 kΩ. Inset displays the schematic of working circuit. Source data are provided as a Source Data file.

Electricity generation performance of md-layer

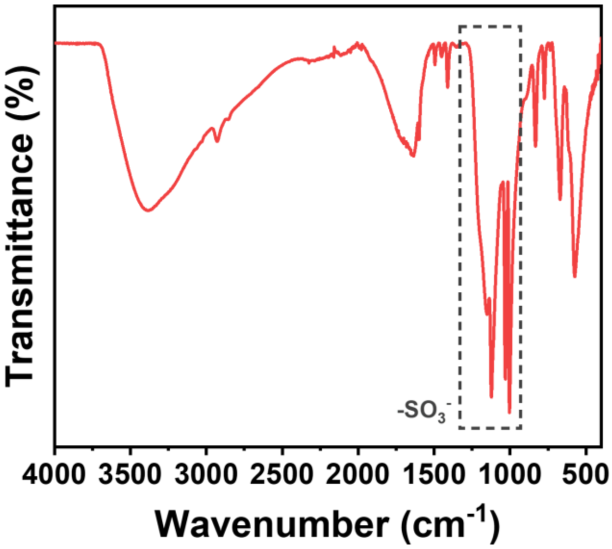

Supplementary Fig. 20 FTIR spectroscopy of the H-PSS membrane. Source data are provided as a Source Data file.

Supplementary Note 5: Analysis of electrical output in md-layer

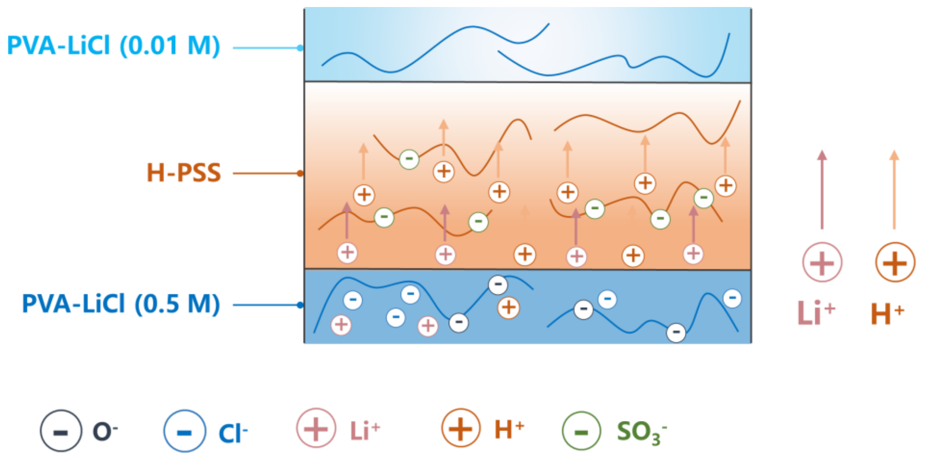

This diagram shows the dissociation and migration of ions in md-layer under water gradient. The PVA-LiCl(*c*) membrane contains hydroxyl (-OH) as well as inorganic salt (LiCl), and the H-PSS membrane contains sulfonic acid group ($-SO_3H$). When moisture enters into the membrane from below, the PVA-LiCl(0.5 M) membrane will be first ionized, forming H^+, Li^+ and Cl^-. Then the mobile H^+ as well as immobile $-SO_3^-$ will come into being in H-PSS membrane, and the immobile polyanion chain segment ($-SO_3^-$) will form the cation channel. Thus the H^+ (orange arrow) and Li^+ (pink arrow) in the PVA-LiCl(0.5 M) membrane will enter into the H-PSS membrane. The diffusion current density is determined by the following equation[5]:

$$J_{\mathrm{Diff}} = -qD\frac{\mathrm{d}c}{\mathrm{d}x}$$

where q, D and *c* represent the carrier charge, diffusion coefficient, and concentration of H^+ as well as Li^+ ions in md-layer.

Diffusion leads to the accumulation of positive charges (H^+, Li^+) on the bottom side and negative charges ($-O^-$, $-SO_3^-$) background on the top side, resulting in a built-in electric field (E, from the bottom up in H-PSS) that will counteract the charge diffusion. Since the

thickness of H-PSS is largely higher than that of PVA-LiCl(c), it is approximately considered that the built-in electric field is formed in H-PSS.

When the moisture infiltrates into the PVA-LiCl(0.01 M) membrane at the top side, a small amount of Li^+, H^+ and Cl^- will form, which can weaken the formation of the built-in electric field to a certain extent. However, more Cl^- were dissociated in PVA-LiCl(0.5 M) film, and the forward migration of Li^+ from PVA-LiCl(0.5 M) membrane is much larger than the reverse migration of Li^+ from PVA-LiCl(0.01 M) membrane. Therefore, the introduction of PVA-LiCl(*c*) has a tendency to increase the built-in electric field in general.

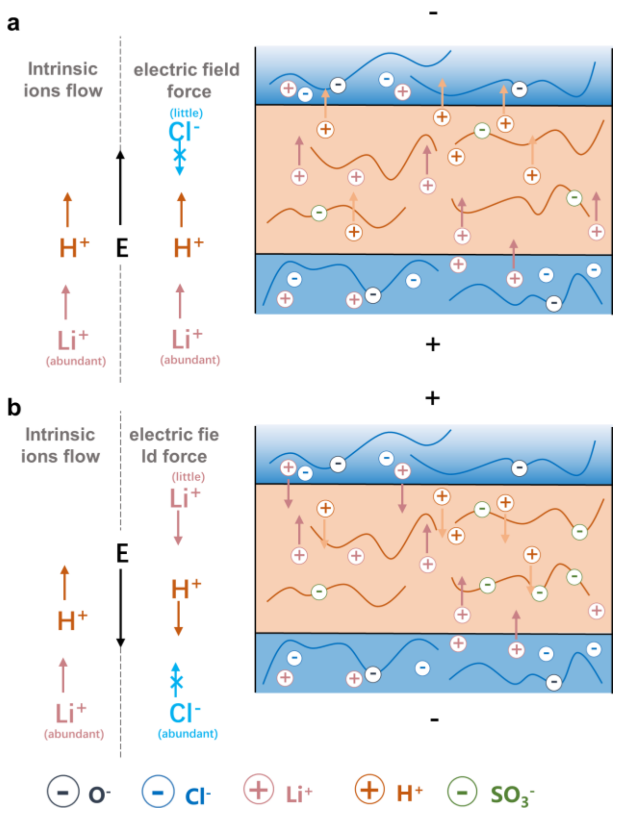

Supplementary Fig. 21 The ions flow direction in md-layer in the electric field force (**a** negative bias **b** positive bias) and the intrinsic ion flow direction determined by ion concentration.

Supplementary Note 6: The electric field accelerates the H^+ as well as Li^+ in PVA-LiCl(0.5 M) migrate into H-PSS and the migration of H^+ in H-PSS when the voltage is from bottom to top (Supplementary Fig. 21a), which is consistent with the moisture induced diffusion direction of Li^+ and H^+ from bottom part into top part of wf-layer above mentioned, while the voltage from top to bottom inhibits the intrinsic migration of H^+ and Li^+ (Supplementary Fig. 21b).

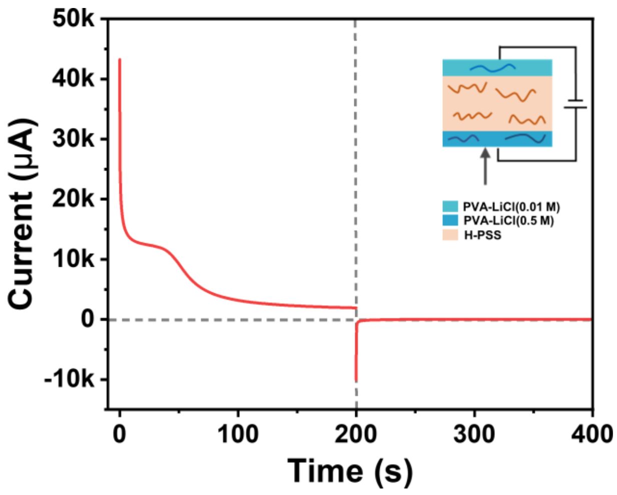

Supplementary Fig. 22 Current-time curve of md-layer device under alternating bias (±1.8 V). Inset displays the schematic of circuit. Source data are provided as a Source Data file.

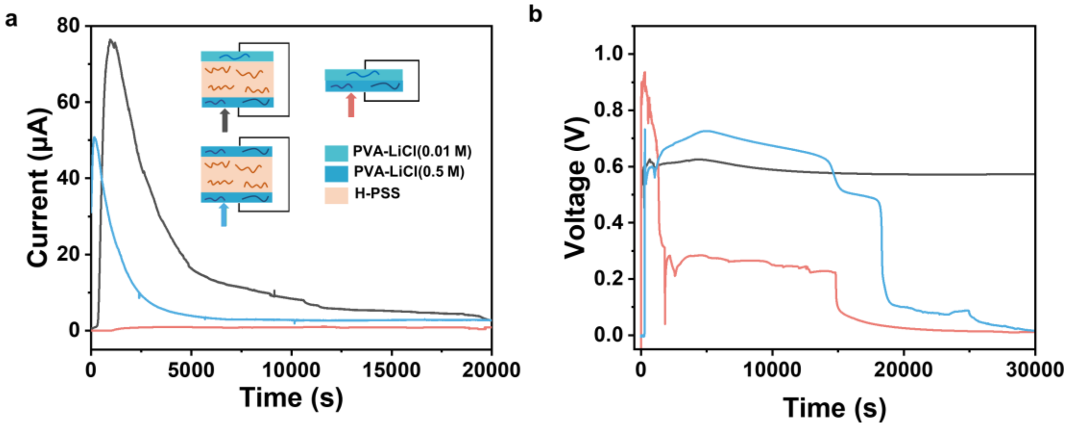

Supplementary Fig. 23 Current-time curves (**a**) and voltage-time curves (**b**) of md-layer (black line), md-layer without H-PSS membrane (red line) and md-layer with the same LiCl concentration (blue line). Insets illustrate the different stacking mode. Source data are provided as a Source Data file.

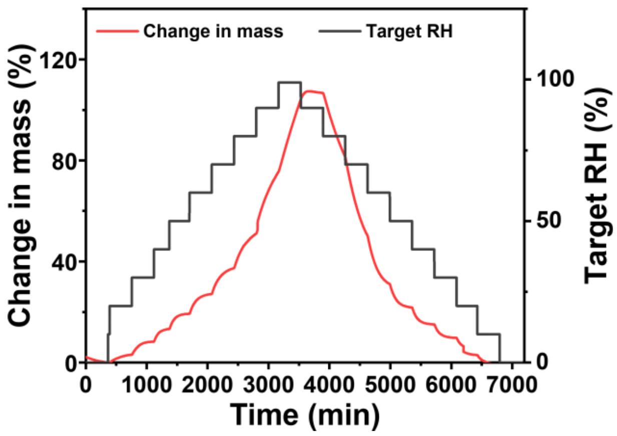

Supplementary Fig. 24 Moisture absorption and desorption kinetics of md-layer at 25 °C.

Source data are provided as a Source Data file.

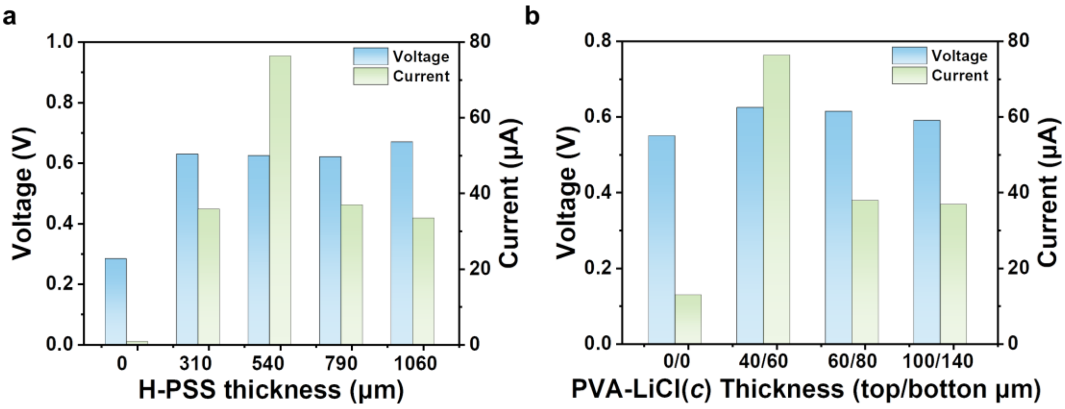

Supplementary Fig. 25 a Voltage and current output of md-layers with different H-PSS membrane thickness. The PVA-LiCl(*c*) thickness were fixed at 40/60 μm. **b** Voltage and current output based on md-layers with different PVA-LiCl(*c*) membrane thickness. The H-PSS thickness were fixed at 540 μm. Source data are provided as a Source Data file.

Supplementary Note 7: When the H-PSS membrane is too thin, the water molecules absorbed by the H-PSS membrane is less and limited mobile ions (H^+) was formed, resulting in lower current ouput[6]. When the H-PSS membrane is too thick, the diffusion pathway for water molecules and mobile ions in the ion selective channel could be too long, inhibiting ions transport in H-PSS[7].

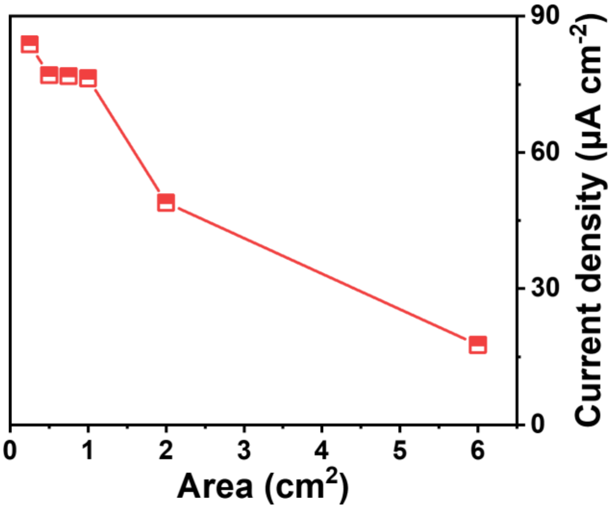

Supplementary Fig. 26 Current density versus area of md-layers. Source data are provided as a Source Data file.

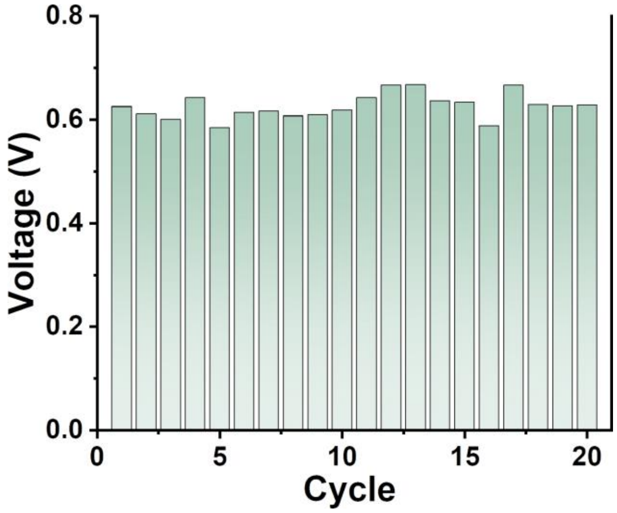

Supplementary Fig. 27 Voltage of md-layer (1 cm × 1 cm) for different cycles. In each cycle, md-layer was dehumidification at 80 ℃, and was put in 90% RH for electricity generation. Source data are provided as a Source Data file.

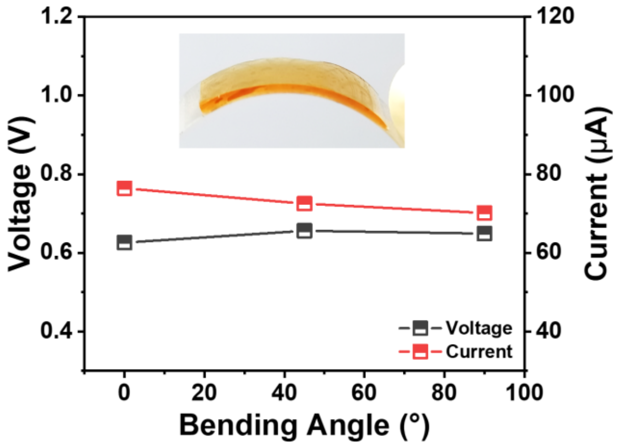

Supplementary Fig. 28 Voltage and current of md-layer (1 cm × 1 cm) under different bending angle. Source data are provided as a Source Data file.

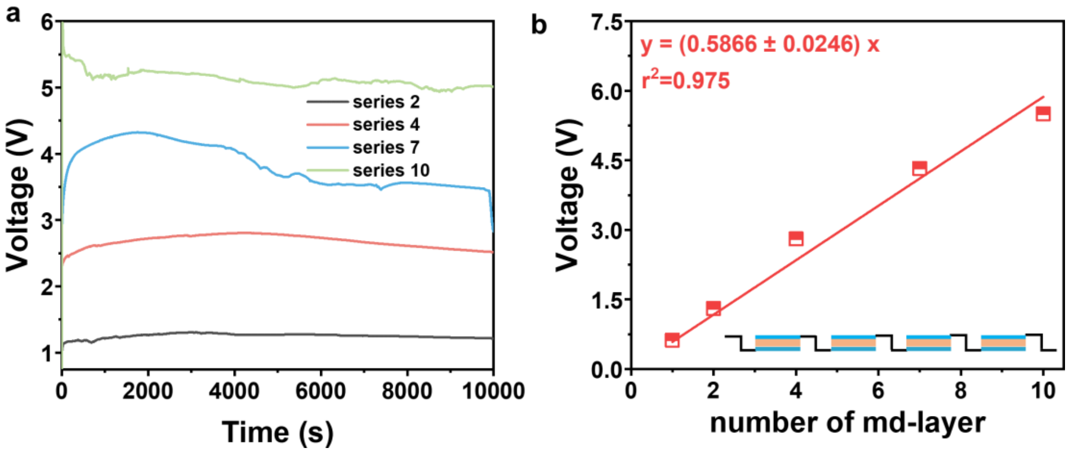

Supplementary Fig. 29 a Voltage-time curves of different numbers of md-layer (1 cm × 1 cm) connected in series. **b** Plot of voltage output with different numbers of md-layer (1 cm × 1 cm) connected in series. Source data are provided as a Source Data file.

Supplementary Note 8: The integration of units is essential to scale up the energy output of device. The generated voltage, which is linear to the number of md-layer, is up to 5.5 V by connecting ten md-layer units in series.

Electricity generation, customized design and applications of mc-WEG

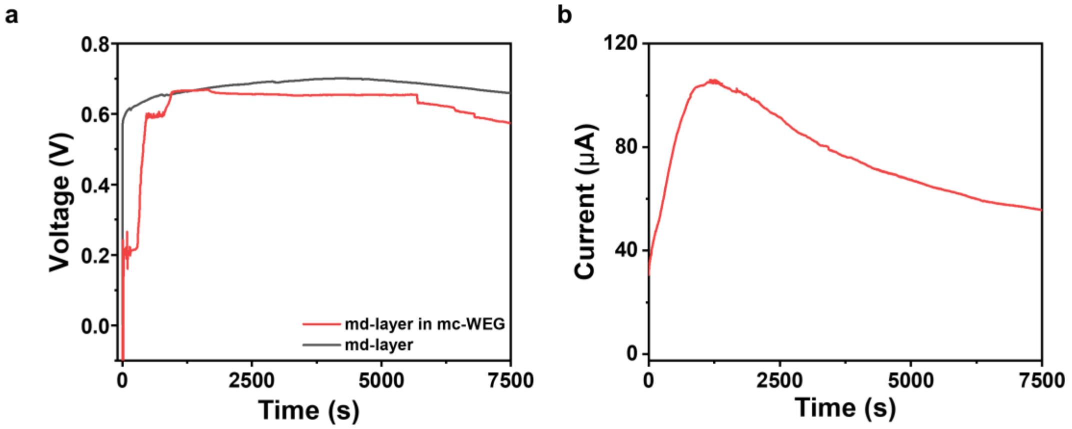

Supplementary Fig. 30 **a** Voltage-time curves of md-layer (3 cm × 2 cm) (black line) and mf-layer in mc-WEG (red line). The ambient humidity is 90% RH. **b** Current-time curve of md-layer (3 cm × 2 cm) in mc-WEG. Source data are provided as a Source Data file.

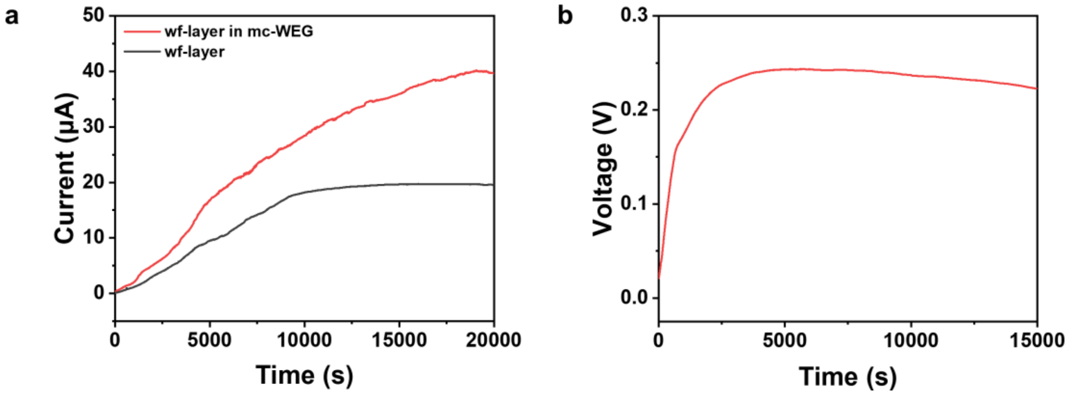

Supplementary Fig. 31 **a** Current-time curves of wf-layer (3 cm × 6 cm) (black line) and wf-layer in mc-WEG (red line). The ambient humidity is 90% RH. **b** Voltage-time curve of wf-layer (3 cm × 6 cm) in mc-WEG. Source data are provided as a Source Data file.

Supplementary Table 1 Output power density of water-enabled electric generators with inert electrodes.

No.	Material	Form of water	P_{output} (mW m^{-2})	Ref
1	Graphene oxide membrane	Moisture	0.018	5
2	Graphene oxide and sodium polyacrylate	Moisture	0.07	8
3	PSS/PVA film	Moisture	7.90	9
4	Protein nanowires	Moisture	0.0405	10
5	Sodium alginate /SiO_2/GO	Moisture	12.00	11
6	Asymmetric hygroscopic of carbon fabric	Moisture	~70	12
7	Ionic polymer Nafion and poly(N-isopropylacrylamide) hydrogel	Moisture	10.14	13
8	Carbon	Liquid	0.053	14
9	Al_2O_3 nanoparticles	Liquid	0.513	15
10	AAO and ionic liquid	Liquid	12.1	16
11	Carbon nanoparticle	Liquid (H_2O)	0.26	3
		Liquid ($CaCl_2$)	0.505	4
12	mc-WEG	Moisture and liquid	91.77	This work

Supplementary Note 9: For devices without power density curves with different electric resistances, the conversion of P_{output} is carried out by the equation[4]: $P_{output} = \frac{V_{oc} I_{SC}}{4}$.

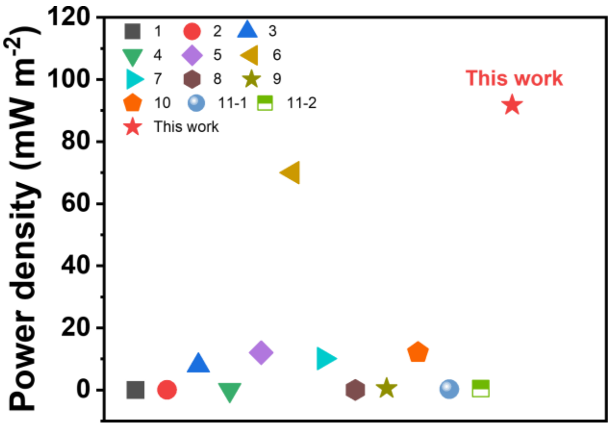

Supplementary Fig. 32 Comparison of area power density between mc-WEG in this work when the two functional layers are output separately and reported water-enabled electric generators. The numbers correspond to the reference numbers in Supplementary Table 1.

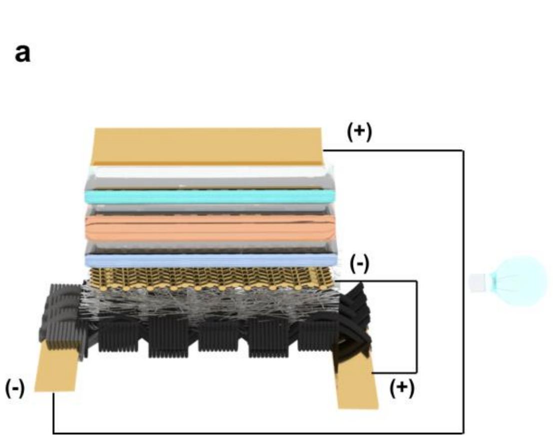

Supplementary Fig. 33 The connection mode between md-layer and wf-layer of mc-WEG when connected in series (**a**) and in parallel (**b**).

Supplementary Note 10: “flexible building block” approach of mc-WEG for customizable energy output

wf-layer and md-layer can be integrated into mc-WEG in the form of “flexible building block” through size control (Supplementary Fig. 34a), space optimization as well as integration design (Supplementary Fig. 34b-d).

Based on the demands of different electronics, when the number of wf-layer in mc-WEG was larger than that of md-layer, taking [1] : (x) (x = 1~4) design for example, the wf-layer building blocks can be designed for a 6 cm × 6 cm size and folded five times to realize the better size matching of the two function layers (Supplementary Fig. 34c). The length of md-layer was 2 cm, and the width was decided by the number of wf-layer (x cm).

When the number of md-layer in mc-WEG was larger than that of wf-layer, taking [x] : (1) (x = 1−4) design for example, the md-layer building blocks can be designed for 0.5 cm × 2 cm size, and the length of wf-layer was 6 cm while the width was decided by the number of md-layer (0.5 × x cm) (Supplementary Fig. 34d).

Meanwhile, different connection design of the inner function layer (in series or parallel) through the design of external circuits can further amplifies the feature of customized output by ulteriorly researching the output performance of mc-WEG unit with more complex connection mode. When used as the power supply source for high current electrical appliances, the number of wf-layer in mc-WEG can be increased appropriately, and more parallel design can be carried out inside. For example, for [1]//(x$^{//}$) (x = 1−4, $^{//}$ indicates that wf-layers in the bottom are connected in parallel, and $^{+}$ indicates series connection) design,

the current improves linearly with the ascending of the number of wf-layer in [1]//($x^{//}$) design (319.75 μA for 4 units, red line in Supplementary Fig. 35a), while the voltage did not change significantly (black line in Supplementary Fig. 35b). Meanwhile, the optimal output resistance also reduces by adding more parallel units in a single mc-WEG units (Supplementary Fig. 36a). Therefore, according to the resistance matching principle, designed [1]//($x^{//}$) unit connected in parallel can be utilized as a supply source for current-driven device (e.g. light-emitting diode (LED)) with low resistance.

When powering appliances that require higher operating voltages, the number of md-layers in mc-WEG can be increased and more series connection can be designed. For example, for [x^+]+(1) (x = 1−4) design, the voltage increases linearly with the augment of the number of md-layer (2.63 V for 4 units, black line in Supplementary Fig. 36c), but the current does not change significantly (red line in Supplementary Fig. 35d). The optimal output resistance also increases by adding more series units (Supplementary Fig. 36b). Thus, designed [x^+]+(1) units connected in series can be utilized as a supply source for voltage-driven device (e.g. field effect transistor) with high resistance.

Moreover, hybrid series and parallel combinations within mc-WEG units can precisely exert similar maximum power density with different output characteristics (voltage and current) and matched resistance. The [1]//($3^{//}$) unit can deliver the maximum power of 664.21 mW m^{-3} at 750 Ω, which corresponding output is 0.24 V and 312.68 μA (Supplementary Fig. 36a), while the [1]//(4^+) unit can exert the maximum power of 602.50

mW m^{-3} at 5.6 kΩ, which corresponding output is 0.71 V and 149.45 μA (Supplementary Fig. 36c).

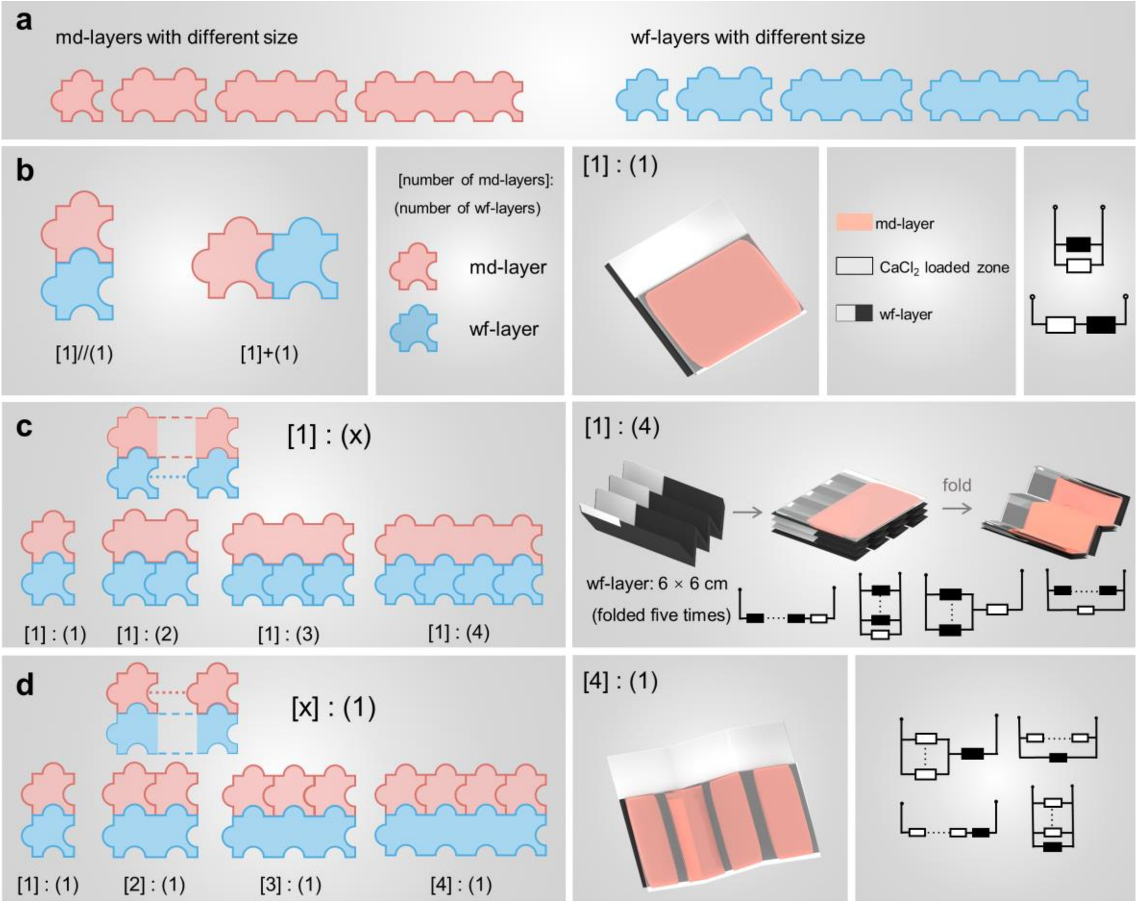

Supplementary Fig. 34 Schematic diagram of the size control, space optimization and integration design of the mc-WEG. Square brackets in literal description denote the number of md-layer, and parentheses denote the number of wf-layer. The size of jigsaw represents the relative size of md-layer and wf-layer in mc-WEG. Circuit diagram in right side represents the partially possible circuit connections under the corresponding combination coefficients. Solid rectangles in circuit diagram represent wf-layer and hollow rectangles represent md-layer. **a** Jigsaw schematic diagram of md-layer and wf-layer with different size. **b** Schematic diagram of [1] : (1) design. When the combination coefficient is [1] : (1), the size of wf-layer (blue jigsaw) is 3 cm × 6 cm, and that of md-layer (pink jigsaw) is 3 cm

× 2 cm. **c** Schematic diagram of [1] : (x) (x = 1−4) design. Middle inset shows the schematic diagram of [1] : (4) unit. When the combination coefficient is [1] : (x) (x = 1−4), the size of wf-layer (blue jigsaw) is 6 cm × 6 cm and folded five times to become 1 cm × 6 cm, and the size of md-layer (pink jigsaw) is x cm × 2 cm. **d** Schematic diagram of [x] : (1) (x = 1−4) design. Middle inset shows the schematic diagram of [4] : (1) unit. When the combination coefficient is [x] : (1) (x = 1−4), the size of wf-layer (blue jigsaw) is x cm ×6 cm, and that of md-layer (pink jigsaw) is 0.5 cm × 2 cm.

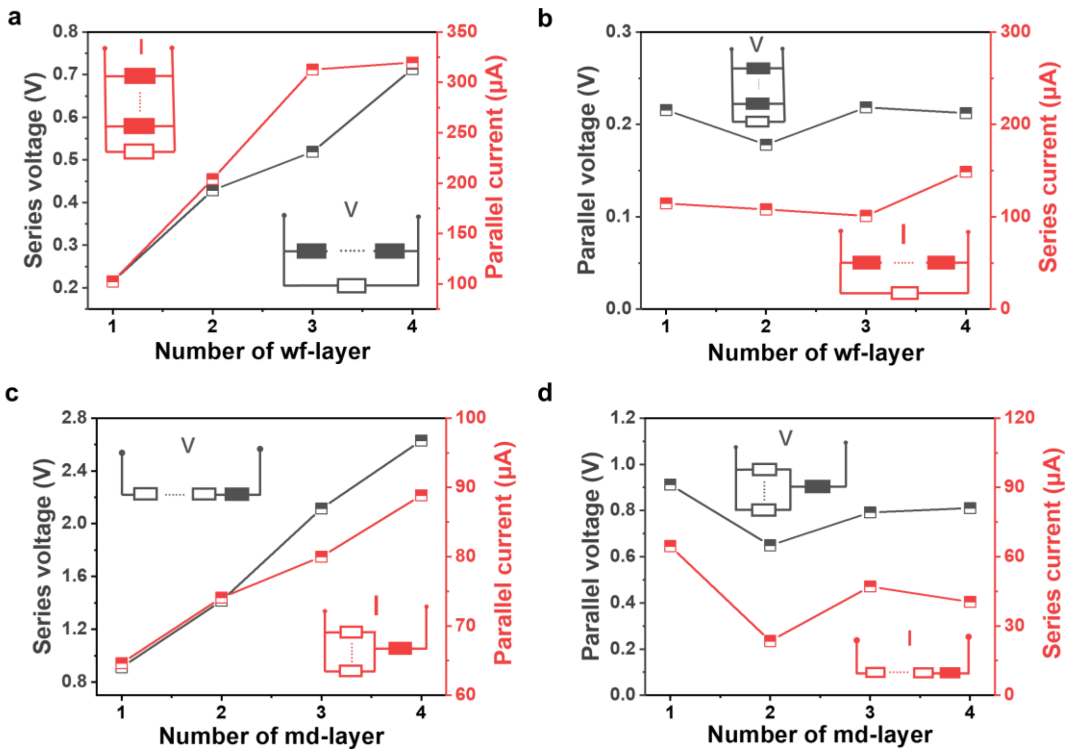

Supplementary Fig. 35 **a** Series voltage of [1]//(x^+) (x = 1~4, black line) and parallel current of [1]//($x^{//}$) (x=1−4, red line). **b** Parallel voltage of [1]//($x^{//}$) (x = 1−4, black line) and series current of [1]//(x^+) (x = 1−4, red line). **c** Series voltage of [x^+]+(1) (x = 1−4, black line) and parallel current of [$x^{//}$]+(1) (x = 1−4, red line). **d** Parallel voltage of [$x^{//}$]+(1) (x = 1−4, black line) and series current of [x^+]+(1) (x = 1−4, red line). The schematic diagram shows the series-parallel mode of the circuit, where solid squares represent wf-layers and hollow squares represent md-layers, the same below. Source data are provided as a Source Data file.

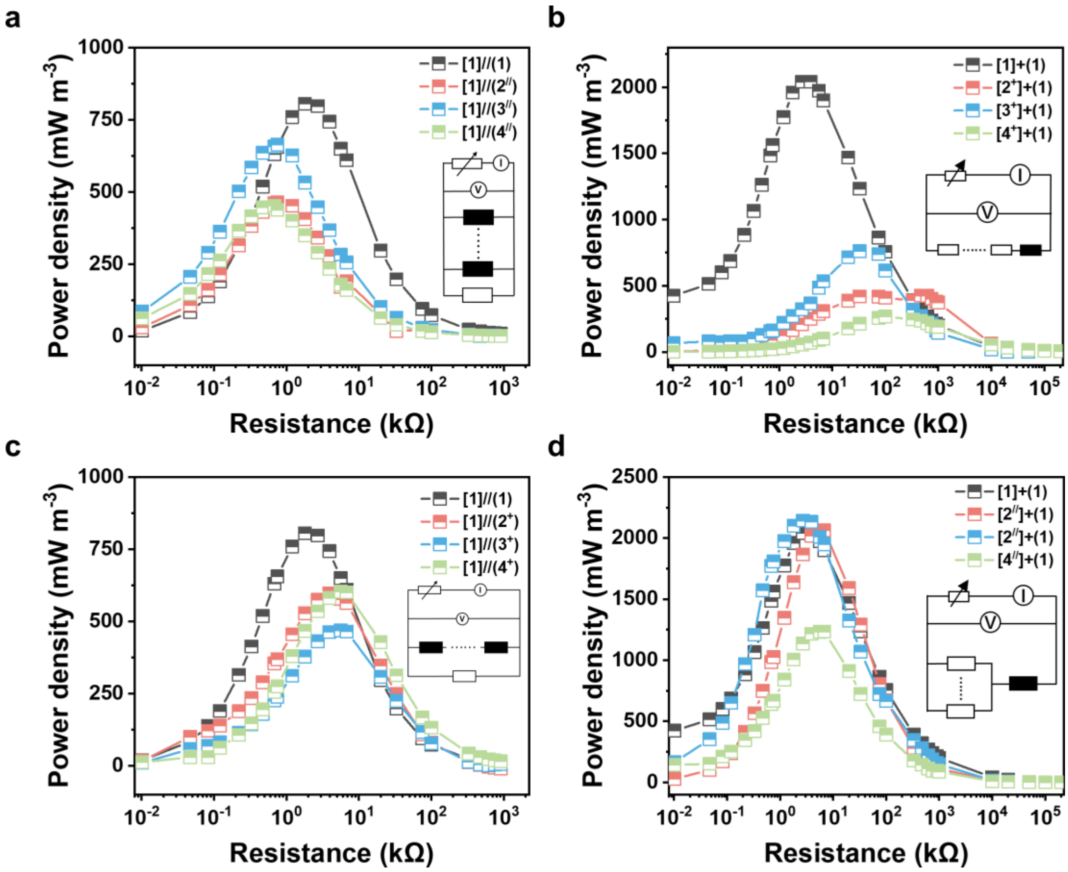

Supplementary Fig. 36 Volumetric power density of [1]//(x$^{//}$) (x = 1−4) (**a**), [x^{+}]+(1) (x = 1−4) (**b**), [1]//(x^{+}) (x = 1−4) (**c**) and [x$^{//}$]+(1) (x = 1−4) (**d**). Source data are provided as a Source Data file.

Supplementary Note 11: Through the series and parallel design of wf-layer and md-layer in mc-WEG, the output voltage and current as well as the internal resistance can be adjusted at the same time, so as to better meet the power demand of different electrical appliances.

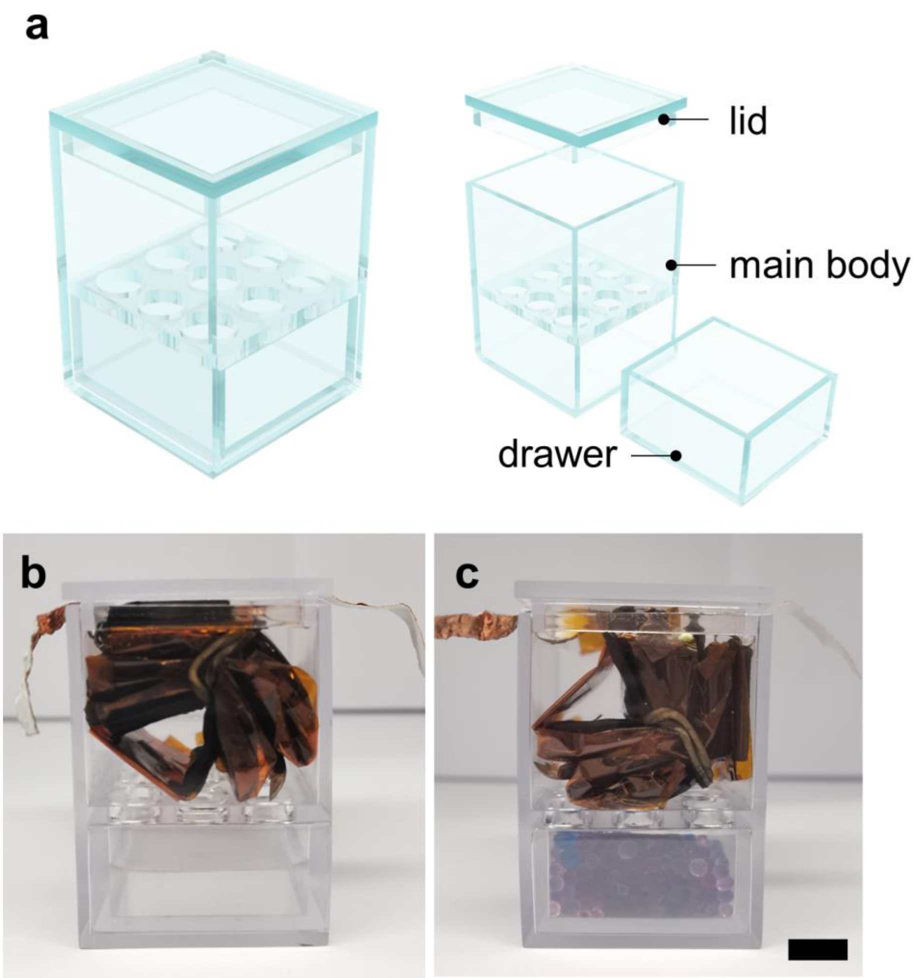

Supplementary Fig. 37 a Schematic diagram of the designed auto-switchable adsorption and desorption generating setup. **b** Photograph of the setup at operative mode. **c** Photograph of setup at preservation mode. Scale bar: 1 cm.

Supplementary Note 12: The designed auto-switchable adsorption and desorption generating setup is consisted of three parts: main body, drawer and lid (**a**). The designed setup can be switched between operative and preservation state for practical application. When the mc-WEG is working, the drawer can be filled with water to create a high humidity environment throughout the box (**b**). When the mc-WEG is at preservation mode, desiccant is placed in the drawer to return the mc-WEG to a dry state and achieve long-term storage (**c**).

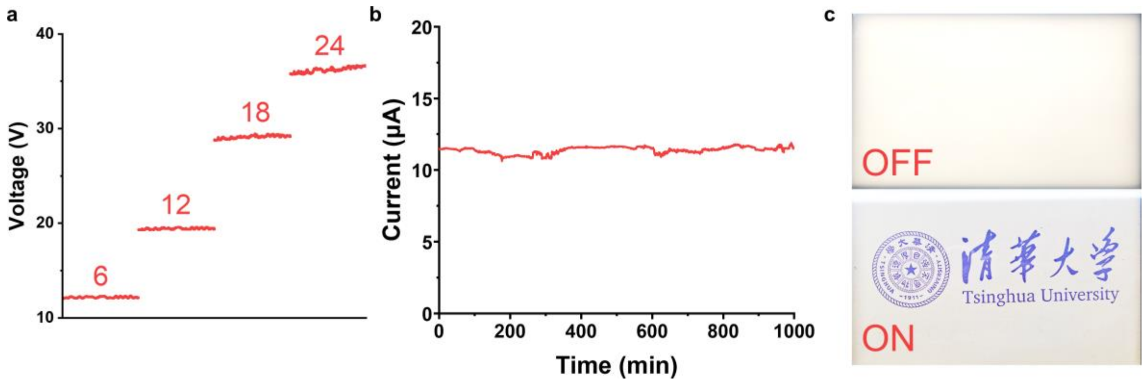

Supplementary Fig. 38 a The relationship between open-circuit voltage and number of mc-WEG_3 connected in series. **b** Current-time curve of twenty-four mc-WEG_3 connected in series. **c** Digital photos of atomized glass. The electrically controlled atomized glass is opaque (upper) when the power is off, which becomes transparent (nether) when twenty-four mc-WEG_3 connected in series act as a power source to energize the glass. Source data are provided as a Source Data file.

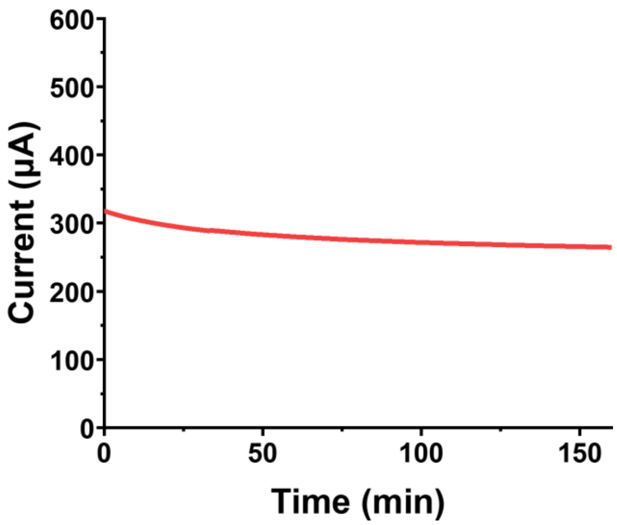

Supplementary Fig. 39 Current-time curve of twenty-two mc-WEG_4 connected in series. Source data are provided as a Source Data file.

Supplementary Table 2 Output power density of water-enabled electric generators with inert electrodes. The volume calculation is based on the whole device.

**No.**	**Material**	**Form of water**	**RH (%)**	$\boldsymbol{P_{output}}$ **(mW m^{-3})**	$\boldsymbol{R_{max}}$	$\boldsymbol{V_{output}}$ **(V)**	$\boldsymbol{I_{output}}$ **(μA)**	**Ref**
1	Graphene oxide membrane	Moisture	25	0.018	~10 MΩ	0.068	0.20	5
2	Graphene oxide and sodium polyacrylate	Moisture	80	0.07	10 MΩ	0.18	0.44	8
3	PSS/PVA film	Moisture	85	7.90	750 kΩ	0.20	0.73	9
4	Protein nanowires	Moisture	45	0.0405	0.7 kΩ	0.24	0.17	10
5	Sodium alginate /SiO_2/GO	Moisture	100	12.00	4 kΩ	0.25	45	11
6	Asymmetric hygroscopic of carbon fabric	Moisture	90	~70				12
7	Ionic polymer Nafion and poly(N-isopropylacrylamide) hydrogel	Moisture	100	10.14				13
8	Carbon	Liquid		0.053	8 MΩ	0.60	0.086	14
9	Al_2O_3 nanoparticles	Liquid		0.513	~6 MΩ			15
10	AAO and ionic liquid	Liquid	95	12.1	5 kΩ			16

11	Carbon nanoparticle		Liquid (H_2O)		0.26				3
			Liquid ($CaCl_2$)		0.505				4
12	mc-WEG	[1]//(1) unit	Liquid and moisture	90	320.94	3.9 kΩ	0.096	24.97	This work
		[1]+(1) unit			133.08	20 kΩ	0.14	7.02	
		[1]//($4^{//}$) unit			452.26	680 Ω	0.21	319.75	
		[4^{+}]+(1) unit			267.86	100 kΩ	2.63	40.40	

Supplementary Note 13: R_{max} is the corresponding electric resistances at the maximum power density. V_{output} and I_{output} are the corresponding voltage and current on the electric resistances at the maximum power density. For devices without power density curves with different electric resistances, the data of R_{max}, V_{output} and I_{output} will be missed, and the conversion is carried out by the equation: $P_{output} = \frac{V_{oc}I_{SC}}{4}$.

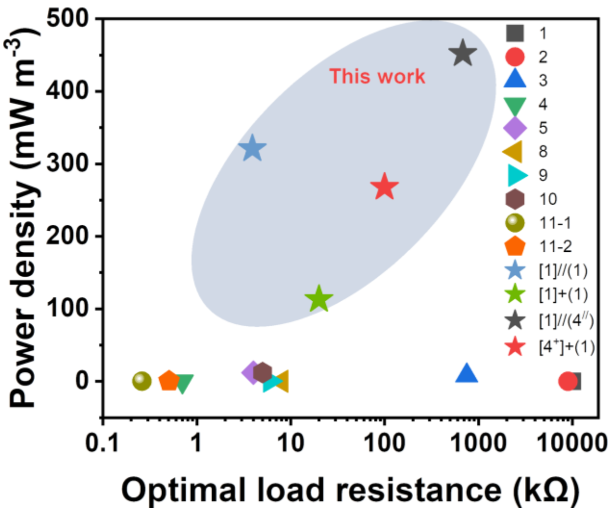

Supplementary Fig. 40 Comparison of output volumetric power density and internal resistance between mc-WEG in this work and reported water induced generators. The numbers correspond to reference numbers in Supplementary Table 2.

References

1 Xu, Y. et al. A one-dimensional fluidic nanogenerator with a high power conversion efficiency. *Angew. Chem. Int. Ed.* **56**, 12940-12945 (2017).

2 Yin, J. et al. Generating electricity by moving a droplet of ionic liquid along graphene. *Nat. Nanotechnol.* **9**, 378-383 (2014).

3 Yun, T. G., Bae, J., Rothschild, A. & Kim, I. D. Transpiration driven electrokinetic power generator. *ACS Nano* **13**, 12703-12709 (2019).

4 Bae, J., Yun, T. G., Suh, B. L., Kim, J. & Kim, I. D. Self-operating transpiration-driven electrokinetic power generator with an artificial hydrological cycle. *Energy Environ. Sci.* **13**, 527-534 (2020).

5 Cheng, H. et al. Spontaneous power source in ambient air of a well-directionally reduced graphene oxide bulk. *Energy Environ. Sci.* **11**, 2839-2845 (2018).

6 Shen, D. et al. Self-powered wearable electronics based on moisture enabled electricity generation. *Adv. Mater.* **30**, 1705925 (2018).

7 Huang, Y. et al. Interface-mediated hygroelectric generator with an output voltage approaching 1.5 volts. *Nat. Commun.* **9**, 4166 (2018).

8 Huang, Y. et al. All-region-applicable, continuous power supply of graphene oxide composite. *Energy Environ. Sci.* **12**, 1848-1856 (2019).

9 Wang, H. et al. Transparent, self-healing, arbitrary tailorable moist-electric film generator. *Nano Energy* **67**, 104238 (2020).

10 Liu, X. et al. Power generation from ambient humidity using protein nanowires. *Nature* **578**, 550-554 (2020).

11 Zheng, S. et al. Continuous energy harvesting from ubiquitous humidity gradients using liquid-infused nanofluidics. *Adv. Mater.* **34**, 2106410 (2022).

12 Tan, J. et al. Self-sustained electricity generator driven by the compatible integration of ambient moisture adsorption and evaporation. *Nat. Commun.* **13**, 3643 (2022).

13 Liu, C. et al. Hydrovoltaic energy harvesting from moisture flow using an ionic polymer–hydrogel–carbon composite. *Energy Environ. Sci.* **15**, 2489-2498 (2022).

14 Xue, G. et al. Water-evaporation-induced electricity with nanostructured carbon materials. *Nat. Nanotechnol.* **12**, 317-321 (2017).

15 Shao, C. et al. Large-scale production of flexible, high-voltage hydroelectric films based on solid oxides. *ACS Appl. Mater. Inter.* **11**, 30927-30935 (2019).

16 Chen, J. et al. Knittable composite fiber allows constant and tremendous self-powering based on the transpiration-driven electrokinetic effect. *Adv. Funct. Mater.* **32**, 2203666 (2022).
