# Supplementary material for: Multistage coupling water-enabled electric generator with customizable energy output
Source: Nat Commun. 2023 Sep 14;14:5702. doi: 10.1038/s41467-023-41371-x (PMC10502115; doi:10.1038/s41467-023-41371-x)
Supplement: Supplementary file 3 — Description of Additional Supplementary Files [file 41467_2023_41371_MOESM3_ESM.pdf]

Description of Additional Supplementary Files

File Name: Supplementary Movie 1

Description: Application of mc-WEG4 pack: lighting a table lamp
